# Current approaches for the fitting and refinement of atomic models into cryo-EM maps using *CCP-EM*

**DOI:** 10.1107/S2059798318007313

**Published:** 2018-05-30

**Authors:** Robert A. Nicholls, Michal Tykac, Oleg Kovalevskiy, Garib N. Murshudov

**Affiliations:** a Structural Studies, MRC Laboratory of Molecular Biology, Francis Crick Avenue, Cambridge CB2 0QH, England

**Keywords:** *REFMAC*5, cryo-EM, model refinement, map blurring, map sharpening, *Divide and Conquer*, *ProSHADE*, symmetry detection

## Abstract

Recent advances in instrumentation and software have resulted in cryo-EM rapidly becoming the method of choice for structural biologists, especially for those studying the three-dimensional structures of very large macromolecular complexes. In this contribution, the tools available for macromolecular structure refinement into cryo-EM reconstructions that are available *via CCP-EM* are reviewed, specifically focusing on *REFMAC*5 and related tools. Whilst originally designed with a view to refinement against X-ray diffraction data, some of these tools have been able to be repurposed for cryo-EM owing to the same principles being applicable to refinement against cryo-EM maps. Since both techniques are used to elucidate macromolecular structures, tools encapsulating prior knowledge about macromolecules can easily be transferred. However, there are some significant qualitative differences that must be acknowledged and accounted for; relevant differences between these techniques are highlighted. The importance of phases is considered and the potential utility of replacing inaccurate amplitudes with their expectations is justified. More pragmatically, an upper bound on the correlation between observed and calculated Fourier coefficients, expressed in terms of the Fourier shell correlation between half-maps, is demonstrated. The importance of selecting appropriate levels of map blurring/sharpening is emphasized, which may be facilitated by considering the behaviour of the average map amplitude at different resolutions, as well as the utility of simultaneously viewing multiple blurred/sharpened maps. Features that are important for the purposes of computational efficiency are discussed, notably the *Divide and Conquer* pipeline for the parallel refinement of large macromolecular complexes. Techniques that have recently been developed or improved in *Coot* to facilitate and expedite the building, fitting and refinement of atomic models into cryo-EM maps are summarized. Finally, a tool for symmetry identification from a given map or coordinate set, *ProSHADE*, which can identify the point group of a map and thus may be used during deposition as well as during molecular visualization, is introduced.

## Abstract 2

*REFMAC*5 and related tools for the refinement of atomic models into cryo-EM reconstructions in *CCP-EM* are reviewed. An upper bound on the correlation between observed and calculated Fourier coefficients is identified, and the practical utility of map blurring/sharpening is discussed. The *Divide and Conquer* pipeline for refining large complexes in parallel, and *ProSHADE* for the identification of symmetries in a given map or coordinate set, are presented.

## 1. Introduction   

Macromolecular X-ray crystallography (MX), nuclear magnetic resonance (NMR) and cryo-electron microscopy (cryo-EM) are the three main experimental techniques that are used to elucidate macromolecular structures in order to answer biological questions. At present, the majority of the structural models deposited in the Protein Data Bank (PDB; Berman *et al.*, 2002 ▸) have been derived using MX (>120 000 models), an order of magnitude more than the second most commonly used technique, NMR (>12 000 ensembles). Although the current proportion of models derived using cryo-EM is comparatively small (>2000), it is becoming the tool of choice owing to the so-called ‘resolution revolution’ caused by rapid advances in instrumentation and software (Faruqi & McMullan, 2011 ▸; Lyumkis *et al.*, 2013 ▸; Kühlbrandt, 2014 ▸; Scheres, 2014 ▸).

Whilst the purpose of these experimental techniques is to answer particular biological questions, our aim is to facilitate this using all available structural information; the purpose of computational tools is to extract as much information as possible from a given data set. Since the information contained in noisy and limited data can be hard to extract, we must develop mathematical and computational tools to help to maximize information extraction in such challenging cases. Of course, there are no computational tools that can replace carefully designed experiments; computation can only aid in experimental design and help to increase the amount of information extracted from the data.

The resolutions quoted for cryo-EM reconstructions vary greatly, and there is a difference in the way in which maps are modelled at different resolutions. If sufficiently high-quality data are available it is now possible to consider *de novo* model building and full atomic refinement (*e.g.* above ∼4 Å, using currently available technology and the current definition of resolution; Rosenthal & Henderson, 2003 ▸). However, at lower resolutions the limited number of observations means that additional prior information, in the form of pre-computed atomic models, may be required. Such initial models would be fitted/morphed into blobs of density. *CCP-EM* (Wood *et al.*, 2015 ▸) contains various tools to facilitate model fitting/refinement, including *DockEM* (Roseman, 2000 ▸), *Choyce* (Rawi *et al.*, 2010 ▸) and *Flex-EM* (Joseph *et al.*, 2016 ▸) for fitting and morphing of the structure at low resolution in cases where only information about the overall shape of the molecule is available, and *REFMAC*5 (Murshudov *et al.*, 2011 ▸) for full atomic model refinement at higher resolutions where at least some bulky side chains are visible. It should be noted that other software tools are available for the fitting and refinement of atomic models into cryo-EM maps, including *DireX* (Schröder *et al.*, 2007 ▸), *MDFF* (Trabuco *et al.*, 2008 ▸), *Cryo-Fit* (Kirmizialtin *et al.*, 2015 ▸), *Rosetta* (Wang *et al.*, 2016 ▸) and *phenix.real_space_refine* (Afonine *et al.*, 2018 ▸).

Experimental data alone are typically insufficient to successfully build and refine an atomic model. Fortunately, our interpretation of incomplete and noisy experimental observations can be improved by using additional sources of information: the stereochemistry of constituent blocks of macromolecules, typical secondary-structure patterns, structures of related macromolecular domains, structural data obtained using different experimental methods *etc.* Information derived from different experiments can be simultaneously co-utilized in order to better address biological questions (Trabuco *et al.*, 2009 ▸; Gong *et al.*, 2015 ▸; Kovalevskiy *et al.*, 2018 ▸). Ideally, staying within realistic and computationally tractable bounds, all available sources of experimental and theoretical information relevant to the molecule of interest should be integrated into one process, with the intention of delivering the best possible structural model for a given state of the molecule.

In both MX and cryo-EM we do not refine against the original data observed in the experiment (in MX the raw data are diffraction-pattern images, while in single-particle cryo-EM the data are two-dimensional particles). Rather we refine against derived data, effectively treating them as if they were experimental observations. Despite the inherent loss of information, this should give reasonable results providing that error estimates are sufficiently accurate.

In MX refinement the ‘observations’ are taken to be the estimated diffraction-spot intensities, which are often converted to structure-factor amplitudes during pre-refinement data processing. Consequently, one needs to solve the ‘phase problem’ either by using prior knowledge about a related structure, *i.e.* molecular replacement (McCoy *et al.*, 2007 ▸; Vagin & Teplyakov, 2010 ▸), or by carrying out additional experiments and determining phases for a substructure (SAD, MAD, SIR *etc.*; Sheldrick, 2015 ▸; Skubák & Pannu, 2013 ▸). Often macromolecules, especially large complexes of exceptional biological interest, fail to form high-quality crystals, resulting in poor diffraction. In such cases, the resulting electron-density maps can be hard to interpret, and additional sources of structural information are often useful when interpreting such poor-quality maps. Indeed, there is an emphasis on the importance of improving phases and the resulting electron-density maps in MX.

This contrasts with atomic model refinement in cryo-EM, in which the ‘observations’ are taken to be the electrostatic potential maps: the outputs of the three-dimensional reconstruction process, which do contain phase information. To date, there have been limited attempts to improve the input maps using information about the current state of the atomic model during refinement (*i.e.* co-refinement of atomic model and three-dimensional reconstructions), although such attempts do include density sharpening by *LocScale* (Jakobi *et al.*, 2017 ▸). Local map quality, *i.e.* local resolution, varies greatly within one and between several reconstructions (Kucukelbir *et al.*, 2014 ▸). Again, the use of additional prior knowledge can help with interpreting the lower resolution parts of such maps that exhibit varying signal-to-noise ratios.

Since the atomic models in both MX and cryo-EM correspond to macromolecules, the prior knowledge used in both of these techniques is essentially the same (Brown *et al.*, 2015 ▸). However, there are qualitative differences between these techniques that affect how model refinement is approached, and the types of problems that are typically encountered include the following.

(i) In MX, crystals belong to one of the possible space groups, with corresponding symmetry operators, and therefore symmetry must be accounted for when calculating the interactions between atoms. In contrast, in cryo-EM we are not dealing with crystals, so we can avoid dealing with crystal properties (for example space groups) and peculiarities (for example twinning). Indeed, there are no crystal contacts, which affects biological interpretation.
(ii) In cryo-EM, there is no fixed unit cell so boundaries are not enforced. Rather, maps are placed in artificial boxes that are large enough to avoid interactions between molecules in neighbouring boxes. These boxes do not have a physical interpretation: they are used for the speed and convenience of calculations only.
(iii) In cryo-EM refinement the ‘observations’ are taken to be the electrostatic potential maps: the outputs of the three-dimensional reconstruction process. In MX refinement the ‘observations’ are taken to be the estimated diffraction-spot intensities, which are often converted to structure-factor amplitudes during pre-refinement data processing.
(iv) In MX the quoted ‘resolution’ is typically the perceived diffraction limit, *i.e.* the resolution of the largest Miller indices that are used. This is itself subjective, and has changed over time as current best practices have evolved (Karplus & Diederichs, 2012 ▸). A different definition of ‘resolution’ is used in cryo-EM, which relates to the signal-to-noise ratio (Rosenthal & Henderson, 2003 ▸). In both scattering techniques the concept of resolution should ideally be reconsidered, accounting for factors such as the strength of the signal-to-noise ratio, the completeness of the data and the nominal resolution (which is related to grid sampling in cryo-EM and the diffraction limit in MX).
(v) Cryo-EM data contain phase information, unlike in MX. Fourier coefficients (both amplitudes and phases) are available when representing cryo-EM electrostatic potential maps in Fourier space, but they are noisy. In contrast, in MX the amplitudes are more accurate but phases are not available.
(vi) In cryo-EM the errors in neighbouring Fourier coefficients are correlated, whereas in MX they are independent.
(vii) Cryo-EM maps represent electrostatic potential, whereas the maps typically viewed corresponding to MX data represent electron density (calculated using phase information from the current state of the model).

Indeed, one major consequence of the ability/inability to observe phase information is in how the maps are calculated. In MX the electron-density maps viewed are calculated using phase information from the current state of the model (*e.g.* 2*mF*_o_ − *DF*_c_ maps), which means that they inherently suffer from model bias. As a result of this, they also have to be updated/recalculated after each round of refinement. This means that the interpretation of these density maps can vary dramatically during the refinement process, particularly at lower resolutions where the data are limited and noisy. In contrast, standard cryo-EM maps are, at present, not calculated using phase information from the current state of the model; they are not updated/recalculated after each round of refinement. Consequently, cryo-EM atomic model refinement can be considered as the problem of fitting into the map whilst ensuring consistency with prior information, ensuring chemical and structural integrity of the model according to our current knowledge of macromolecular structures.

In this contribution, we review the tools available for macromolecular structure refinement into cryo-EM reconstructions that are available *via CCP-EM* (Burnley *et al.*, 2017 ▸). Specifically, we focus on the program *REFMAC*5 (Murshudov *et al.*, 2011 ▸), noting that other refinement software and suites employ similar technologies (see, for example, Afonine *et al.*, 2013 ▸). These tools were originally designed with a view to refinement against MX diffraction data, but the same principles are applicable to refinement against cryo-EM maps (Murshudov, 2016 ▸; Brown *et al.*, 2015 ▸). The importance of phases is considered, as well as the potential utility of replacing inaccurate amplitudes with their expected values. Issues that should be contemplated when performing refinement against cryo-EM reconstructions are emphasized, notably an upper bound on the correlation between the atomic model and observed map, based on the Fourier shell correlation between half-maps, and how care should be taken when choosing which level of blurred/sharpened map to refine the model against. Features important for the purposes of computational efficiency are discussed: the necessity for appropriate box-size selection, and the *Divide and Conquer* pipeline, which enables the refinement of large complexes to be computationally tractable by refining different parts of the model in parallel. For completeness, other relevant tools to aid atomic model refinement in *REFMAC*5 and *Coot* (Emsley *et al.*, 2010 ▸) are discussed, notably the use of prior information and tools with a wider radius of convergence to facilitate the refinement of highly displaced regions of a model. Finally, a new tool for the detection of rotational symmetries in atomic models and maps, *ProSHADE*, is presented. It should be emphasized that the recommendations and features described in this contribution relate to the current state of existing software tools; in future it would be advantageous for improved techniques and tools to be developed and implemented.

## 2. The importance of phases   

It is well known that for the purpose of map calculation the phases of structure factors are more important than their amplitudes. To analyse this statement, we can consider the correlation between the current and ‘ideal’ maps. Specifically, since correlations calculated in real and reciprocal space are equivalent, we consider the Fourier shell correlation (FSC) calculated over all structure factors,
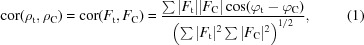
where a subscript C denotes the current map and a subscript t denotes the ‘true’ (or ‘ideal’) map, and ρ represents map density with corresponding structure factors *F* with amplitudes |*F*| and phases φ. If we consider structure factors in a narrow resolution range then we can express the FSC in terms of the normalized amplitudes |*E*|. Then, under the assumptions that the reciprocal-space points are sufficiently dense and that the distribution of Fourier coefficients in shells reflects the ‘true’ distribution, we can express the FSC as the expected value of the weighted cosine of phase differences,
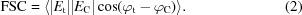



It is clear that for ‘good’ maps the FSC would be higher; if we had two maps, and were able to calculate the FSC between these maps and the corresponding ‘true’ map, then we would prefer the one that exhibited the higher FSC. An important, if perhaps obvious, point to note is that if the phases are random and the amplitudes are exact then the FSC will be zero.

Now consider the limiting case where the phases are exact and the amplitudes are random. Under the assumption that the amplitudes come from a Wilson distribution, we have
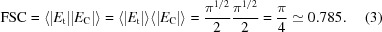



However, if we replace all amplitudes by their expected value (in a given bin), *i.e.* |*E*_C_| = 〈|*E*_C_|〉, then we instead obtain
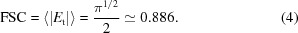



Consequently, given that this value is greater than 0.785, if the structure factors have little or no information about the structure under analysis then it might be better to replace the observed amplitudes with their expected values (for further discussion in the context of MX, see Nicholls *et al.*, 2017 ▸).

It must be emphasized that the above analysis is valid for map calculation only, *i.e.* not for use in model refinement. Indeed, for the refinement of model parameters we must have the conditional probability distribution of observed data given model parameters. It seems sensible to use all observations during refinement provided that errors are estimated accurately.

## 3. Correlation between atomic model and observed maps   

In practice, we are not able to directly calculate the FSC between the current and ‘ideal’ maps. However, we are able to calculate the correlation between the observed and calculated coefficients: cor(*F*_o_, *F*_c_). Furthermore, if half-data sets are available then we are also able to calculate the FSC between the two half-data sets: FSC_1/2_. Therefore, we are able to consider the relationship between FSC_1/2_ and cor(*F*_o_, *F*_c_).

Let us assume that we have observations, and that the errors in the observations are additive: *F*_o_(*s*) = *F*_T_(*s*) + *F*_n_(*s*), where a subscript o denotes observations, a subscript T denotes the ‘true’ image and a subscript n denotes noise. Let us further assume that we have Fourier coefficients *F*_c_(*s*) calculated from the atomic model. In the absence of overfitting we can also assume that there is no correlation between calculated Fourier coefficients and noise in the data. The correlation between observed and calculated Fourier coefficients in narrow Fourier shells can thus be calculated as
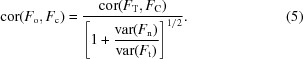



If we have half-data sets, then (see Appendix *A* [App appa])
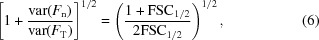
resulting in
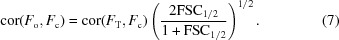



This relationship only holds if there is no correlation between the signal *F*_c_(*s*) and noise in the data. However, when the atomic model is refined against observed data then fitting, at least partially, into the noise is unavoidable. Consequently, we can use this relationship to infer an upper limit on the correlation between observed and calculated Fourier coefficients: it should never exceed [2FSC_1/2_/(1 + FSC_1/2_)]^1/2^. For example, if the FSC_1/2_ is 0.5 then the correlation between the observed and calculated Fourier coefficients should not exceed (2/3)^1/2^ ≃ 0.82. Were a correlation higher than this value observed then further investigation would be required.

## 4. Blurring and sharpening   

In cryo-EM, variability of the reconstructed molecule owing to heterogeneity of the sample and computational inaccuracies during the reconstruction causes blurring of the signal in the map. Map sharpening has been used to counter over-blurred maps, resulting in features in the map being revealed (Brunger *et al.*, 2009 ▸; Nicholls *et al.*, 2012 ▸), noting that other approaches towards map modification have been employed with a similar objective both in the context of MX (Afonine *et al.*, 2015 ▸) and cryo-EM (Jakobi *et al.*, 2017 ▸; Terwilliger *et al.*, 2018 ▸). Conversely, if the map has been over-sharpened then blurring may be required. Reconstruction programs perform post-processing in order to deblur or sharpen the resultant map. However, even if the noise variance is constant within the reconstructed map, owing to the varying mobility of the molecule over space it can be expected that the signal-to-noise ratio will also vary over space. The deblurring parameter should depend on the signal-to-noise ratio, so a single parameter value may not be sufficient for all parts of the map. Consequently, different parts of the map may require different levels of sharpening/blurring for optimal interpretation, and thus may still need additional sharpening or blurring in order to achieve optimal results.

In MX, map calculation is usually performed as a separate step after refinement, so sharpening/blurring does not affect refinement. However, in cryo-EM sharpening/blurring is performed after reconstruction but before model building, so it may directly affect the behaviour of refinement. Consequently, careful thought is required as to the appropriate level of sharpening/blurring in order to achieve optimal refinement results. Furthermore, the appropriate levels of sharpening/blurring required for model building may differ from those required for refinement.

**Figure 1:**
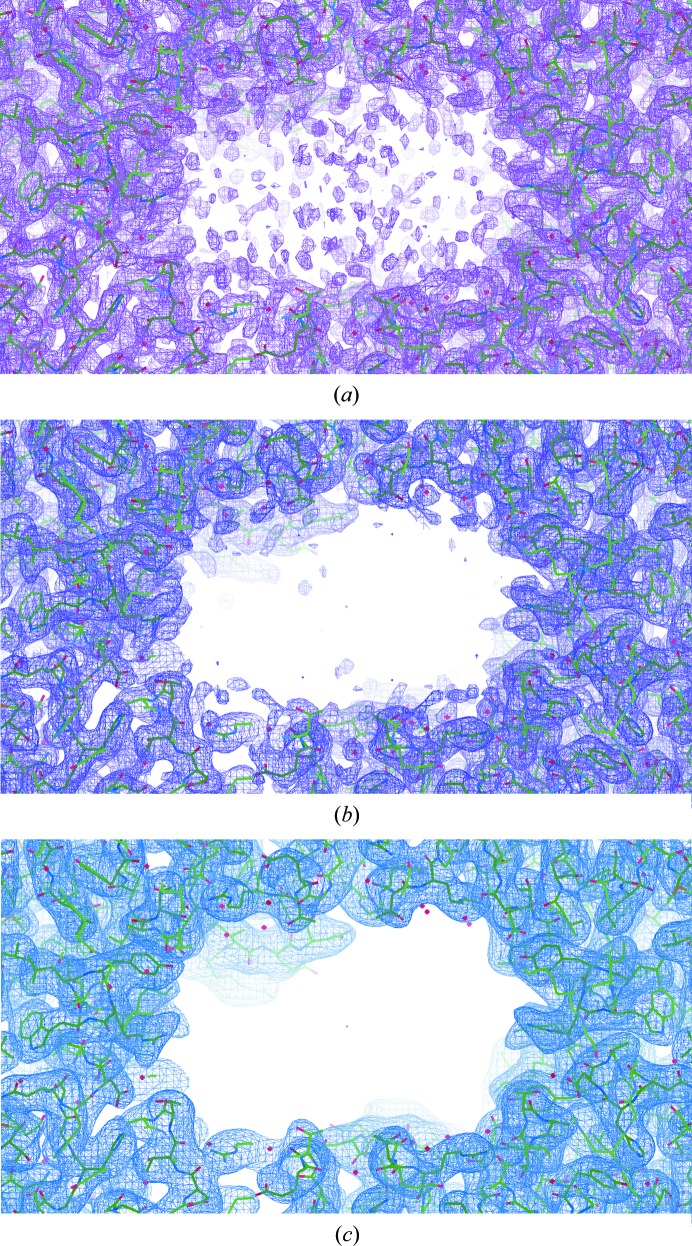
The effect of map blurring on cryo-EM reconstructions. Electrostatic potential maps and atomic models corresponding to the structure of β-­galactosidase (PDB entry 5a1a, EMDB entry EMD-2084; Bartesaghi *et al.*, 2015 ▸). (*a*) The original map is very noisy, suggesting that it is over-sharpened. (*b*) Blurring the map using a *B* value of 40 Å reduces the noise, appearing to make it largely disappear, although the features (signal) in the map remain clear. This produces a clearer map that it will be easier to refine the atomic model against. (*c*) Applying further blurring (*B* value of 100 Å) does not help: it causes more of the noise to disappear, but also causes structural details to be lost (for example regarding side chains).

Refining against an over-blurred map will have a negative affect on the atomic model, as it may increase the overall *B* value beyond reason. As a low-pass filter, map blurring reduces high-frequency noise whilst reducing finer structural details (for example side chains). Conversely, over-sharpening is inadvisable; as it exacerbates high-frequency noise in the map and increases the series-termination effect, it may mask out the signal and result in an uninterpretable map. In the extreme, the visual distinction between protein and solvent regions would diminish. Consequently, the selection of appropriate levels of blurring/sharpening is important (Fig. 1 ▸).

**Figure 2:**
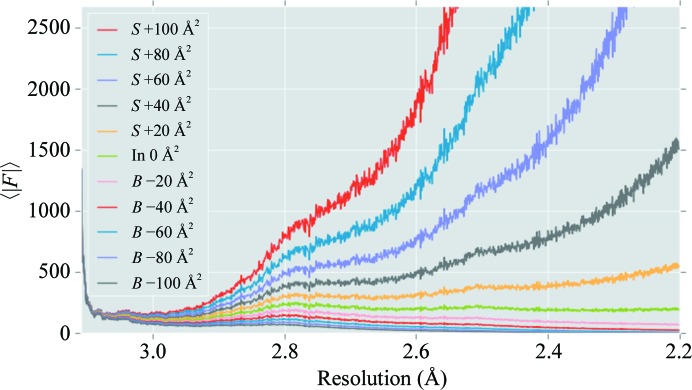
Plot of average structure-factor amplitude against resolution to aid the selection of the optimal blurring/sharpening *B* value, as shown in the *CCP-EM* GUI (Burnley *et al.*, 2017 ▸). The data correspond to a cryo-EM reconstruction of β-galactosidase (PDB entry 5a1a, EMDB entry EMD-2084; Bartesaghi *et al.*, 2015 ▸). Different levels of map blurring and sharpening are shown, using an array of *B* values from −100 Å (blurring) to +100 Å (sharpening) in increments of 20 Å. For the optimally blurred/sharpened map, the average amplitude should decay to zero at the ‘high-resolution limit’ (above which the data contain no information about the true structure). For high levels of map sharpening, the average amplitude persistently increases with resolution: this indicates over-sharpening. For high levels of map blurring, the average amplitude approaches zero rapidly prior to reaching the high-resolution limit, resulting in a loss of high-resolution information. In this case, the optimal average level of blurring over the map is around 40 Å (subjectively determined by manual inspection of the plot), in agreement with the maps shown in Figs. 1 ▸ and 3 ▸.

Selecting appropriate blurring/sharpening *B* values may be facilitated by analysing how the average structure-factor amplitude varies with resolution: this should gradually decay with increasing resolution, yet if the map is over-sharpened then it will instead increase (Fig. 2 ▸).

**Figure 3:**
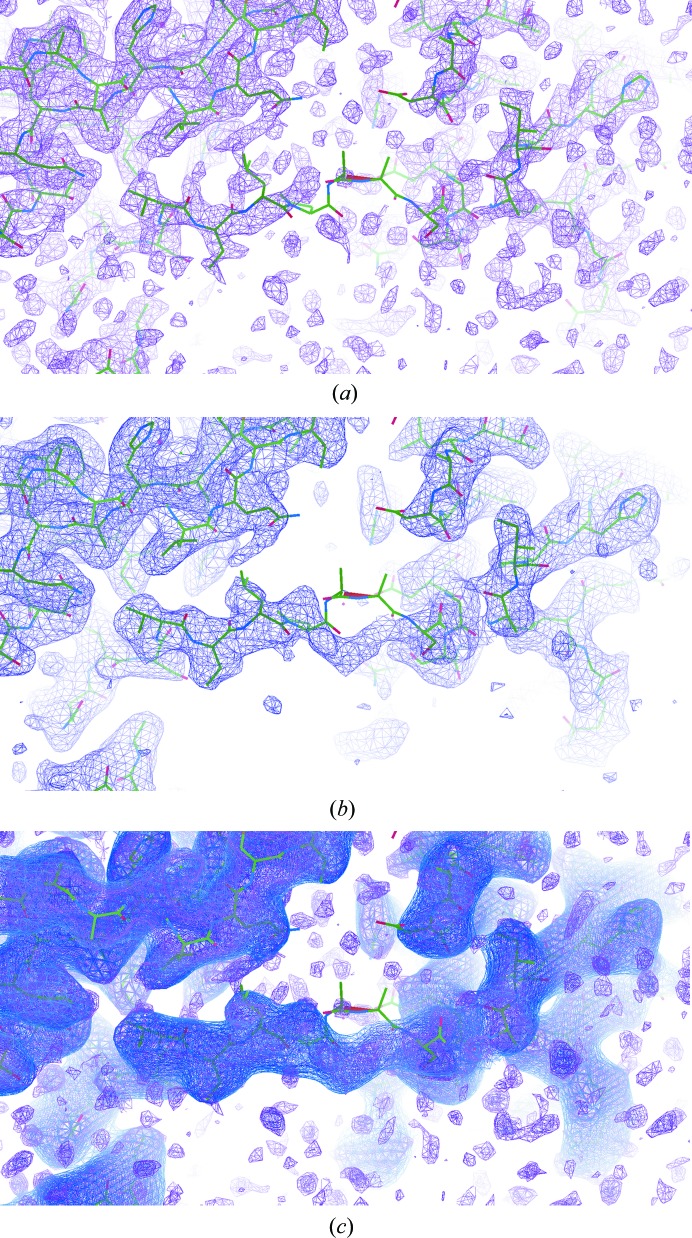
The utility of simultaneously viewing multiple maps using various levels of map blurring/sharpening. Electrostatic potential maps and atomic models corresponding to β-galactosidase (PDB entry 5a1a, EMDB entry EMD-2084; Bartesaghi *et al.*, 2015 ▸) using different levels of map blurring. (*a*) In the original/default map, noise is visible in the solvent regions and there is little backbone density visible in the exposed region around residue 732 in chain *A*. (*b*) Blurring the map using a *B* value of 40 Å reduces noise and reveals features in the map. This suggests incorrect modelling of the backbone in this region and indicates where the model backbone should be positioned. (*c*) Simultaneously viewing an array of maps with different levels of sharpening and blurring further increases map interpretability. Here, maps corresponding to blurring *B* values of 0, 20, 40, 60, 80 and 100 Å are displayed. In this case, it is evident how the backbone should be traced (from the more blurred maps), and at the same time it is possible to interpret finer structural details such as the orientations of side chains (from the sharper maps).

However, considering the behaviour of the average structure-factor amplitude only gives an overall picture, whereas different localized regions of the map may require different levels of blurring/sharpening. Consequently, owing to map heterogeneity it is advisable to work with multiple blurred and sharpened maps. Doing so can allow the accurate building of atomic models, accounting for overall shape and allowing the backbone to be traced, as well as finer structural details such as the position and orientation of side chains. Indeed, it is often useful to view multiple maps with differing levels of sharpening/blurring simultaneously in order to maximize visual interpretability; this strategy can help to gain more information than can be obtained by looking at a single map alone (Fig. 3 ▸).

An array of blurred/sharpened maps can be output by *REFMAC*5 and loaded into *Coot* automatically using the *CCP-EM* GUI (Burnley *et al.*, 2017 ▸). However, note that this is for gaining intuition by visual inspection of the maps, and that at present *REFMAC*5 will only use one map with one level of blurring/sharpening when performing the actual refinement. Indeed, care must then be taken to ensure that the appropriate level of sharpening/blurring is used for atomic model refinement.

Analysis of the distribution of *B* values in an atomic model can also help in deciding an appropriate level of blurring/sharpening to be applied to the map prior to refinement. Note that it does not make physical sense for a large number of atomic *B* values to cluster around a small value (or become negative). However, if the map has been excessively over-sharpened then the atomic *B* values can become stuck, clustering around a minimum value; in such cases it may not be possible to recover the distribution of *B* values by reblurring. If all *B* values are high then this could indicate that the map has been over-blurred, in which case it is possible to further sharpen the map.

However, it should be noted that the refinement *R* factors change (decrease) as the blurring *B* value increases. This is systematic behaviour and does not necessarily imply a model of increased quality. Indeed, overall *R* factors depend on the overall *B* value (Brown *et al.*, 2015 ▸). The FSC is much more stable under different levels of blurring, but there is still an effect. This means that it is not appropriate to compare refinement statistics (especially *R* factors) between models if the maps have been subjected to different levels of blurring.

## 5. Selected tools for atomic model refinement   

One important similarity between MX and cryo-EM is that the data derived using both techniques come from scattering experiments. In both cases there is typically high-resolution information loss. Sufficient quality lower resolution information is typically obtained (for example regarding the overall shape and position of macromolecular domains) but the quality of the data degrades as the resolution increases, inhibiting the observation of finer structural details. Thus, for both techniques it is necessary to somehow account for the loss of high-resolution information that could not be observed sufficiently well during the experiment.

Many of the software tools that have been developed and established for MX refinement were designed to deal with this type of problem: refining atomic models in the presence of high-resolution information loss. Owing to this inherent similarity between the problems of MX and cryo-EM model refinement, it has been possible to repurpose many of these software tools for use in the refinement of models derived using high-resolution cryo-EM.

There has been debate as to whether refinement should be performed in real space or reciprocal space; both approaches have advantages and disadvantages. In real space, refinement can be performed locally, which has advantages for computational speed and parallelization. However, refinement in reciprocal space allows errors in Fourier coefficients to be accounted for more accurately. Although the errors in neighbouring Fourier coefficients are correlated, they are less correlated than those between proximal points in real space. Furthermore, the degree to which errors are correlated will depend on the nature of the underlying data (*i.e.* on the reconstruction methods). There is a common misconception that the original data are in real space. Reconstructions are typically performed in Fourier space using the projection-slice theorem, as for example in *RELION* (Scheres, 2012 ▸) and *Frealign* (Grigorieff, 2007 ▸), before the reconstructed maps are subsequently calculated in real space. Alternatively, three-dimensional reconstruction may be performed in real space using back-projection, noting that this results in a large correlation radius of errors in real space. These two procedures in real and reciprocal space are mathematically equivalent; in both cases refinement should be equivalent (apart from details of implementation). The community would benefit from clarification about best practice on this controversial topic; thus proper analysis will be required in the future. For discussion of the similarity of real- and reciprocal-space refinement, details of the reciprocal-space maximum-likelihood refinement target used in *REFMAC*5 for cryo-EM and the utility of half-maps for purposes of validation, see Brown *et al.* (2015 ▸) and Murshudov (2016 ▸).

### 5.1. Prior information   

The prior probability distribution must minimally contain information about bond lengths and angles: basic chemical information, such as ‘ideal’ bond lengths and angles, is usually employed universally. As the resolution decreases, longer and longer range information is needed to complement the data. The use of information about torsion angles, secondary structures, domains and intra-domain interactions might be required. *B*-value restraints are also used, as it is generally expected that neighbouring atoms will have similar *B* values in regions where modelled atoms are positioned sufficiently accurately (Nicholls *et al.*, 2017 ▸).

**Figure 4:**
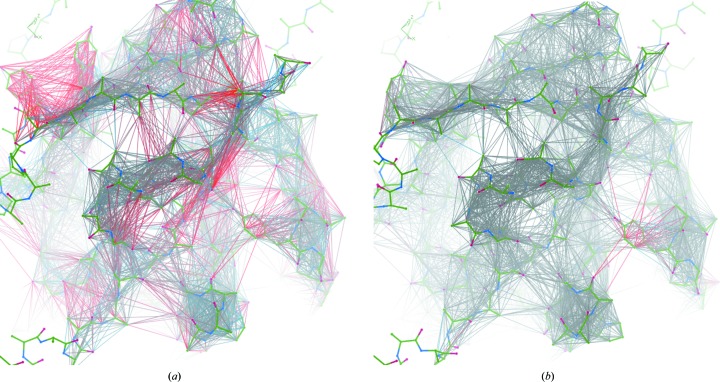
External restraint visualization and usage during parallelized full-chain real-space atomic model refinement in *Coot*. In order to mimic a ‘typical’ stage of the model-building and refinement process in which the model is complete yet the conformational geometry is suboptimal, the atomic model corresponding to the RAD51 filament (PDB entry 5jzc, EMDB entry EMD-8183; Short *et al.*, 2016 ▸) was subjected to simple re-refinement, causing overfitting into the map. External restraints were generated by *ProSMART* using a homologous structural model derived using MX (PDB entry 1n0w; Pellegrini *et al.*, 2002 ▸) as a reference. In *Coot*, the colouring of interatomic vectors representing restraints indicates local structural conservation; consistent interatomic distances are coloured grey, whilst those that are substantially longer/shorter in the target than in the reference model are coloured red/blue. (*a*) The re-refined model of 5jzc is shown, along with the interatomic distance restraints that were generated using 1n0w. From observing the restraint colouring it is evident that there are substantial local conformational differences between the two structural models despite the sequence homology. (*b*) Performing full-chain real-space refinement of the model in *Coot* results in the local conformation of the model becoming much closer to that of the high-resolution homologue, as indicated by the increased quantity of interatomic vectors coloured grey. Regions containing restraints coloured red/blue indicate persisting differences between the target and reference models. Such regions are indicative of either true differences between the target and reference structures (as is the case in Fig. 5 ▸), suboptimal restraint weighting parameters or errors in the model that require manual resolution. Regardless, the presence of any such regions would warrant closer inspection.

Additional sources of prior knowledge relevant to macromolecules include structural information from reference models of known homologues, knowledge about secondary structures, hydrogen-bonding patterns *etc*. This information is encapsulated in the form of external restraints, which may be generated using software tools such as *ProSMART* (Nicholls *et al.*, 2014 ▸) and *LibG* (Brown *et al.*, 2015 ▸), and used during refinement by *REFMAC*5 (Nicholls *et al.*, 2012 ▸) and *Coot* (Fig. 4 ▸). Such structural information has also been exploited in a similar way by other software packages (Headd *et al.*, 2014 ▸, 2012 ▸; Schröder *et al.*, 2010 ▸; Sheldrick, 2015 ▸; Smart *et al.*, 2012 ▸). Additionally, prior information encapsulating local conformational conservation can be exploited, keeping local interatomic distances similar to those in the starting atomic model. Jelly-body restraints have proven to be particularly useful regularisers as they do not inject any information that was derived externally (for discussion, see Nicholls *et al.*, 2017 ▸, 2013 ▸). They are often used to help modelled regions refine into a map in a concerted fashion (having a wide radius of convergence) as well as to ensure the stability of refinement during all stages of the process.

**Figure 5:**
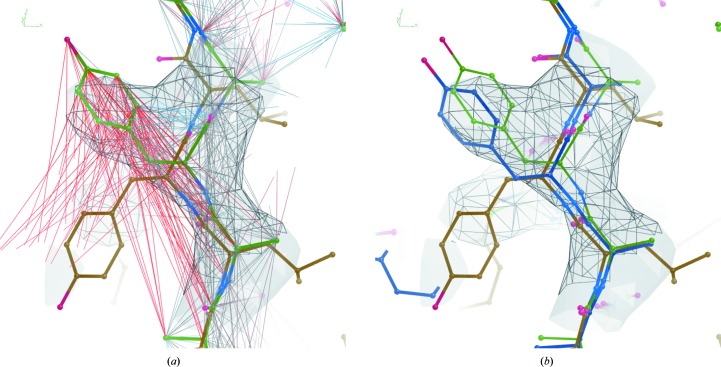
Robustness to outliers when performing atomic model refinement using external restraints. The re-refined atomic model corresponding to the RAD51 filament (PDB entry 5jzc, EMDB entry EMD-8183; Short *et al.*, 2016 ▸), as shown in Fig. 4 ▸(*a*), is displayed coloured green. External restraints were generated using *ProSMART* using the homologous structural model derived using MX (PDB entry 1n0w; Pellegrini *et al.*, 2002 ▸) as a reference. The homologous model 1n0w is shown coloured brown superposed onto 5jzc. (*a*) The red colouring of the restraints reflects differences in the rotameric state of the aligned functional tyrosine between the target and reference models (Tyr293 in 5jzc, Tyr301 in 1n0w). Map density is clearly visible; this side chain is oriented acceptably in the original model, so it would be undesirable for the external restraints to pull this side chain into the conformation observed in the homologous model. (*b*) The model after real-space re-refinement with external restraints in *Coot* (as shown in Fig. 4 ▸ *b*) is coloured blue. Owing to the use of robust estimation, which restricts the influence of outliers, the tyrosine side chain retains its original conformation rather than being pulled out of the map and into the rotameric state observed in the homologous model. However, the geometry of the backbone in this region is improved, adopting a similar conformation to the homologous model as appropriate. Following such injection of prior structural information, the typical protocol would then be to perform full-model refinement using *REFMAC*5 with jelly-body restraints, which would allow the model to relax into the density whilst ensuring refinement stability.

One of the problems of using long-range information as prior knowledge is the inherent dependence on the structural environments of the molecules. Consequently, special care must be exercised when using such information: well known techniques such as robust estimator functions (Huber, 2011 ▸) are used in order to improve the application of long-range information derived from known structures (Fig. 5 ▸).

### 5.2. Box-size selection   

Unlike in MX, in cryo-EM there is no fixed unit cell. Boundaries are not enforced by the experiment, and thus they have to be chosen. The selection of an appropriate box size is important from a computational perspective: choosing a larger box size for a given resolution would result in the requirement for finer sampling in Fourier space in order to avoid a loss of map information owing to interpolation. In turn, using a finer sampling would dramatically slow Fourier space-based optimization procedures.

It is typical for the map output from the three-dimensional reconstruction to have a box size that is larger than necessary for use in model refinement. Consequently, it is often necessary to reduce the box size prior to refinement. This is available as an option in the *REFMAC*5 section of the *CCP-EM* interface, allowing the box size to be determined (reduced) automatically by creating a mask of a given radius around the model. By default, a 3 Å hard mask around the atomic coordinates is used at present.

### 5.3. *Divide and Conquer*   

Attempting to fit and refine atomic models into cryo-EM reconstructions corresponding to very large complexes can be a computational challenge. Complexes consisting of several hundreds of protein chains, with molecular weights of over 10 MDa, are now being encountered in practical application (see, for example, Zhang *et al.*, 2017 ▸). Dealing with such cases can be a technical challenge, comprising many reconstructions of partially overlapping maps, extending to high resolution (in the range 3–4 Å), split across multiple files.

Such large complexes cannot be refined as a whole, owing to both computer memory limitations and the computational complexity associated with increasing map sizes. To refine such huge structures, we split the map and model into smaller more manageable portions, refine them separately and then put them back together at the end. Specifically, this procedure performs the following.

(i) It analyses all interchain contacts in the original structure (which may comprise multiple files).
(ii) It identifies all possible substructures consisting of (1) a target chain and (2) all surrounding chains. The number of resultant substructures is thus equal to the number of chains.
(iii) For each substructure, a map is prepared that covers the substructures. This map may be composed from several different overlapping input maps.
(iv) *REFMAC*5 is then used to refine each substructure (in parallel, either locally or on a computing cluster).
(v) The full complex is then re-assembled from each of the refined chains.

**Figure 6:**
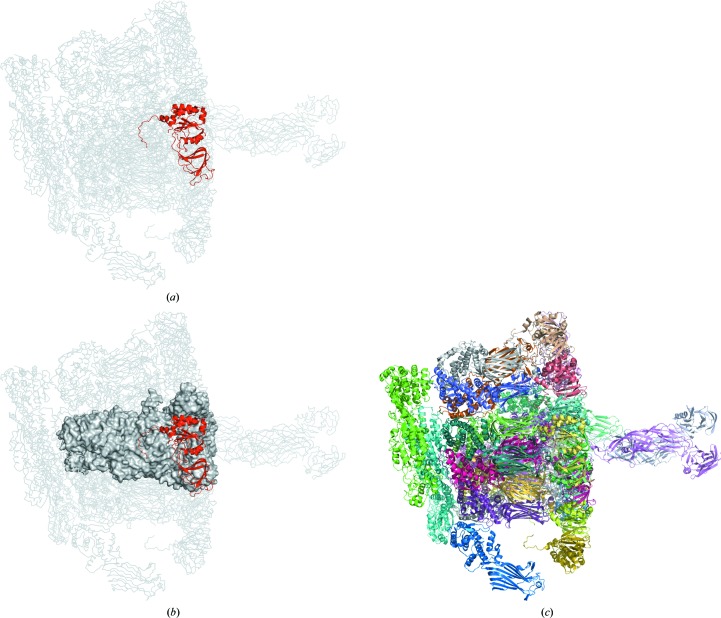
The *Divide and Conquer* procedure illustrated using a model of a rotavirus particle (PDB entry 4v7q, EMDB entry EMD-5199; Settembre *et al.*, 2011 ▸) visualized using *PyMOL* (Schrödinger). (*a*) The input macromolecule is split into individual chains. (*b*) For each chain, the adjacent contacting chains are identified and included for the refinement of the target chain. Each such substructure is masked in order to identify an appropriate box size for use in refinement. (*c*) After parallel refinement of each individual substructure, all of the target chains are extracted and recombined into the macromolecular complex.

This approach, termed *Divide and Conquer*, is available on request and has already been successfully used for structure determination (Zhang *et al.*, 2017 ▸). *Divide and Conquer* will be available as an option in the *CCP-EM* interface that is intended to expedite and parallelize the refinement of huge models containing hundreds of chains (Fig. 6 ▸).

### 5.4. Other relevant tools for model refinement in *REFMAC*5 and *Coot*   

Following fold recognition and the building/placement of an initial atomic model, it is often the case that the model is located out of the density. In such cases, the model will need to be optimally positioned before detailed refinement can be performed. For this type of application, it is necessary to use a refinement technique with a sufficiently large radius of convergence. In simple cases this can be achieved using rigid-body refinement (available in various packages; see, for example, Afonine *et al.*, 2009 ▸). To account for conformational differences between the initial starting model and the map, it is often more appropriate to use restrained refinement with jelly-body restraints in *REFMAC*5 (Murshudov *et al.*, 2011 ▸). These restraints keep the local conformation of the molecule intact, whilst allowing groups of atoms (for example secondary structures or domains) to move in a concerted fashion. This helps to avoid local minima, increasing the radius of convergence of refinement (Nicholls *et al.*, 2013 ▸), noting that other suites employ comparable or different approaches to address this type of problem (Schröder *et al.*, 2007 ▸; Wang *et al.*, 2016 ▸).

In cases where encountering local minima during refinement is unavoidable, the use of other algorithms or manual intervention may be required. *Coot* (Emsley *et al.*, 2010 ▸) includes refinement tools designed for this purpose that help to improve the local fit to density. Specifically, Jiggle Fit helps to appropriately position and orient the atomic model (rigid-body fitting), and Model Morphing (Terwilliger *et al.*, 2013 ▸) allows localized regions to be fitted into the map by applying local shifts to the atomic model, whilst ensuring robustness so as to avoid geometric distortions (Brown *et al.*, 2015 ▸). These tools are particularly useful when dealing with highly displaced regions of the model (for example macromolecular domains). Typically, such procedures should then be followed by full model refinement in *REFMAC*5 using jelly-body restraints in order to stabilize refinement. It should be noted that flexible molecular-dynamics-based fitting/refinement is available as an alternative approach to morphing (for example *Flex-EM*; Joseph *et al.*, 2016 ▸).

Following initial fitting of the model, atomic ‘real-space’ refinement can be performed within *Coot*, allowing the fit of localized regions of the atomic model to be optimized, combined with manual intervention. In cryo-EM maps, the signal-to-noise ratio is often such that additional restraints are needed to stabilize the model. Such restraints, for example those generated by *ProSMART* or *LibG*, can be imported into *Coot* for use during real-space refinement. These interatomic distance restraints can be displayed for purposes of visual­ization, providing feedback regarding the consistency between the restraints and the current state of the model (see Figs. 4 ▸ and 5 ▸).

Whilst real-space refinement in *Coot* is most typically used to refine individual residues or localized regions, it is sometimes desirable to refine larger regions (for example whole chains). This has recently become computationally tractable owing to parallelization of real-space refinement in *Coot*.

## 6. Rotational symmetry   

Many protein structures are known to have rotational symmetry, with over 38% of the entries in the PDB having some form of rotational symmetry assigned. The symmetry information is frequently used in structure solution as well as to decrease the storage requirements by storing only the asymmetric portion of the structure and all symmetry operators required to generate the full structure. While the symmetry is usually known when the structure is being solved, there is a lack of a simple tool for rotational symmetry detection in either electron-density maps or atomic models.

### 6.1. Rotational symmetry detection using rotation function   

The developed tool *ProSHADE* can take either an atomic model or a density map as an input; the atomic models are converted into a theoretical density map using the Clipper library (Cowtan, 2003 ▸) before subsequent processing. Density maps are then mapped onto a set of concentric spheres, and each sphere is decomposed using the spherical harmonics decomposition. The spherical harmonic coefficients are used to compute the rotation function integral over the radius (Navaza, 1994 ▸), which is then used to compute the inverse Fourier transform in the space of rotations SO(3); both the SO(3) transform and the spherical harmonics decomposition are computed using the SOFT library (Kostelec & Rockmore, 2007 ▸).

The inverse SO(3) Fourier transform space may be parameterized using Euler angles α, β and γ as indices, with the values being the cross-correlations between the structure and a rotated version of itself. The highest value is therefore obtained for angles α = β = γ = 0, but any structure with internal rotational symmetry about the origin will also have a peak representing each rotation which produces high cross-correlation between the original and rotated structures. As the cyclic symmetry (denoted *C*_*n*_, where *n* is the order of rotational symmetry) is defined as a point group for which any rotation by 2π/*n* radians about the symmetry axis does not change the shape, it is clear that any such symmetry will have a signature set of peaks detectable in the inverse SOFT map.

It then follows that by analysing the peaks in the inverse SOFT map, it should be possible to determine the position and order of rotational symmetry, thus detecting any *C*_*n*_ symmetry present in the structure. Once the *C*_*n*_ symmetries have been detected, it is further possible to determine the presence of any dihedral symmetries (*D*_*n*_) owing to their property of consisting of two cyclic symmetries *C*_*n*_ and C_2_ with perpendicular axes of symmetry. Similarly, tetrahedral symmetry (*T*) has the characteristic property of having two *C*_3_ symmetries with an axis angle of cos^−1^(1/3) ≃ 1.23 rad, while the icosahedral symmetry (*I*) can be detected by finding *C*_5_ and *C*_3_ symmetries with an angle between them of cos^−1^(5^1/2^/3) ≃ 0.73 rad.

**Figure 7:**
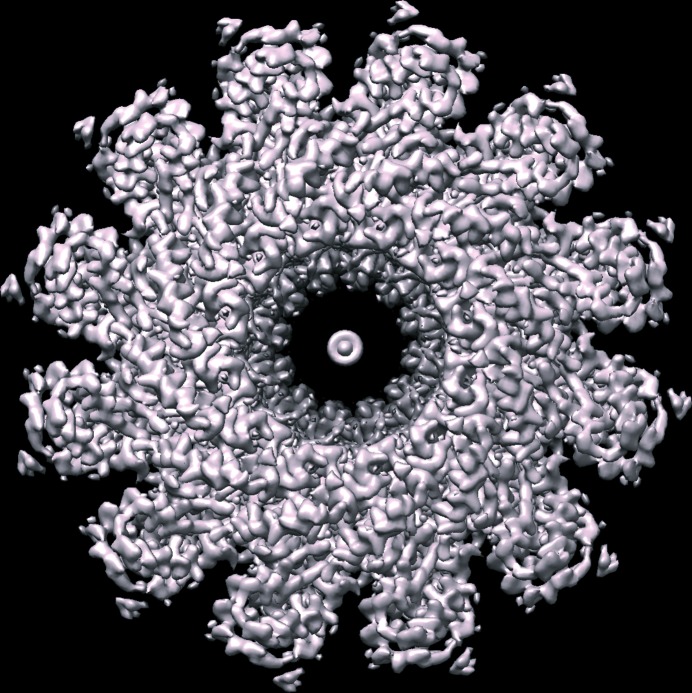
Symmetry detection using *ProSHADE*. The map corresponding to a bacteriophage T4 portal protein (PDB entry 3ja7, EMDB entry EMD-6324; Sun *et al.*, 2015 ▸) visualized using *UCSF Chimera* (Pettersen *et al.*, 2004 ▸). *ProSHADE* reports the correct *C*12 symmetry, with the symmetry axis along the vector (0.02, 0.02, 1.00) with an average cross-correlation between the original structure and the structures rotated along this symmetry axis of 0.443. All symmetry elements present for this instance of the *C*12 symmetry group are reported, along with the list of alternative symmetries that are also present in the structure (*C*6, *C*4, *C*3 and *C*2) and the corresponding rotation-function peak heights.

The aforementioned rules for detecting the *D*, *T* and *I* symmetries are sufficient to find the appropriate symmetries of the structure. However, to find all of the symmetry operators the complete point groups need to be generated. Nonetheless, the two point-group elements listed in the aforementioned rules for each of the *D*, *T* and *I* symmetries are sufficient to generate the complete point groups; this follows from the fact that the two finite cyclic rotation generators are independent. Therefore, by using the inverse SOFT map-based approach, reliable detection and complete point-group element generation is possible. An example of symmetry detection using *ProSHADE* is shown in Fig. 7 ▸.

## 7. Discussion   

In this contribution, we describe several tools available from *CCP-EM* (Wood *et al.*, 2015 ▸) and *CCP*4 (Winn *et al.*, 2011 ▸). We anticipate that the *Divide and Conquer* algorithm will become useful in facilitating the refinement of large molecules with potentially multiple maps corresponding to multiple focused reconstructions. We emphasize the importance of selecting appropriate levels of map blurring/sharpening, which may be facilitated by considering the behaviour of the average map amplitude at different resolutions, and the utility of simultaneously viewing multiple blurred/sharpened maps. These tools are available from within the *CCP-EM* interface (Burnley *et al.*, 2017 ▸).

Model building using cryo-EM maps poses special problems, and is often the most time-consuming part of the cryo-EM data-interpretation process. Several of the tech­niques available in *Coot* (Emsley *et al.*, 2010 ▸) have successfully been used by structural biologists (see, for example, Casañal *et al.*, 2017 ▸) for this purpose, notably Jiggle Fit for positioning and orienting the atomic model, Model Morphing to allow localized regions to be fitted into the map and the use of externally derived restraints that can be visualized as well as applied during refinement to aid stability and/or improve geometry. Recent efforts towards refinement parallelization have resulted in the ability to refine larger regions of the model concurrently.

We present a tool for symmetry identification from a given map or coordinate set: *ProSHADE*. Whilst it is likely that map symmetry is accounted for during three-dimensional reconstruction, this information is often lost. Ideally, this information should be carried from reconstruction to the deposition of maps and atomic models. *ProSHADE* can identify the point group of a map, and thus may be used during deposition as well as during molecular visualization. Further details will be provided elsewhere. *ProSHADE* is available upon request, with the intention of distributing it *via* *CCP*4 and *CCP-EM* in the future.

We also discuss the importance of phases, and the potential utility of replacing poor-quality observations with their expectations. Specifically, with random amplitudes but exact phases, the correlation between the current and ‘true’ maps is ∼78.5%. In contrast, when phases are random the FSC will be zero, irrespective of the accuracy of the amplitudes. We thus infer that phases are much more important than amplitudes (a fact that has been known for a long time). Furthermore, if we replace random amplitudes with their expectations then the FSC increases to ∼88.6%. Thus, if the structure factors have little or no information about the structure under analysis, then there may be utility in replacing the observations with their expected values.

More pragmatically, we demonstrate that there is an upper limit of [2FSC_1/2_/(1 + FSC_1/2_)]^1/2^ on the correlation between observed and calculated Fourier coefficients, expressed in terms of the FSC between two half-data sets. Should correlations be observed above this limit, further investigation would be warranted.

### 7.1. Future perspectives   

Recent advances in cryo-EM have resulted in this method rapidly becoming the method of choice for structural biologists, especially for those studying the three-dimensional structures of very large macromolecular complexes. For the last 50 years or so, macromolecular crystallography, especially that using X-ray scattering, has been the main technique for structure elucidation. Consequently, there is a wealth of accumulated experience and knowledge of this technique. It is tempting to re-use tools developed for X-ray crystallography for cryo-EM data analysis and modelling. Although some of these tools could well be transferred between these techniques legitimately, there are some significant differences that should be accounted for. Since both techniques are used to solve the structures of macromolecules, tools encapsulating prior knowledge about macromolecules can easily be transferred. These include the generation and use of restraints describing constituent blocks of macromolecules (Long *et al.*, 2017 ▸) and the transfer of local conformational information between homologous macromolecular structures (Nicholls *et al.*, 2012 ▸; Kovalevskiy *et al.*, 2018 ▸). Moreover, since both are the result of particle scattering, most of the Fourier-based techniques can be used for analysing both types of experimental data.

However, there are significant differences between these experimental techniques and these need to be accounted for if the objective is to derive the ‘best’ atomic model using noisy observations.

(i) Cryo-EM produces images of whole molecules, and as a consequence this includes information about the phases of the Fourier transformation. In contrast, most crystallographic calculations are designed to recover phase information lost during the experiment. We need to develop new techniques for cryo-EM map improvement and refinement. If such new techniques are to be developed then they must aim to improve the Fourier coefficients overall, not just the phases (Murshudov, 2016 ▸).
(ii) Observational errors in Fourier coefficients for crystallographic data can be assumed to be independent. However, this cannot be assumed for cryo-EM reconstructions. By considering the nature of reconstruction, it can be shown that noise will always be correlated between neighbouring points in the map, as well as in Fourier space. In the future, the joint conditional probability distributions of observed data given (atomic) model parameters will need to account for this correlation.
(iii) The concept of ‘resolution’ is problematic in crystallography as well as in cryo-EM: a problem that has been highlighted by Wlodawer & Dauter (2017 ▸) and re-emphasized in the context of cryo-EM by Wlodawer *et al.* (2017 ▸). In crystallography the resolution is defined by the highest Miller index present in the data file. This does not account for incompleteness or the signal-to-noise (standard deviation of observational noise, observed sigma) ratio in the data. Moreover, since phases are used in map calculation, when defining resolution the quality of the phases and therefore the quality of the maps should be accounted for. In cryo-EM the resolution is defined using the FSC between half-data set reconstructions; resolution is considered as a solution of the equation FSC(*s*_max_) = 0.143. However, this approach has been criticized (van Heel & Schatz, 2017 ▸). This definition does not account for the variation in the FSC or the correlation between noise in the data. Moreover, since the mobility of atoms varies over the whole molecule, it can be expected that the signal-to-noise ratio will depend on the mobility of molecules in particular regions. In future, we will need to develop a better definition of ‘resolution’ that will account for all of the above properties.
(iv) The atomic form factors corresponding to X-ray and electron scattering are different; in X-ray crystallography observations correspond to the electron density of atoms, whereas in electron diffraction and cryo-EM observations correspond to the electrostatic potential. There is a table of atomic form factors for electron scattering (Peng *et al.*, 1996 ▸) that is used widely by many popular programs. There is also a relationship between X-ray and electron scattering form factors *via* the Mott–Bethe formula (Kirkland, 2010 ▸), which is used by *REFMAC*5 (Murshudov, 2016 ▸). However, these tables do not accurately account for the chemical type-dependence of scattering, or for screening owing to the realignment of surrounding charges. These factors will need to be accounted for in the future.

There are many other problems that require further attention, including (i) the refinement of very large molecules against very large maps, (ii) the validation of derived atomic models against observed data, (iii) the full automation of model building using similar and/or invariant substructures, (iv) optimal difference map calculation between observed maps and between observed and calculated maps, accounting for all sources of error along with the potential correlations between them, and (v) accurate atomic form factors for electron scattering.

## Appendix A Relationships between different Fourier shell correlations

Relationships between Fourier shell correlations (FSCs) calculated in different scenarios have been described elsewhere (see, for example, Rosenthal & Henderson, 2003 ▸; Karplus & Diederichs, 2012 ▸), including the FSC between two half-data reconstructed images, the FSC between two full-data reconstructions and the FSC between observed and true Fourier coefficients. To make statements and calculations that are more precise, we will use the following assumptions: (i) there is no correlation between signal and noise, (ii) there is no correlation between the noise in the two half-data reconstructions and (iii) the noise in the Fourier shells represents the noise in the data, *i.e.* the distribution of the noise for all Fourier coefficients in sufficiently narrow Fourier shells is the same as the distribution of the noise for any single Fourier coefficient taken from this shell. We will use the following notation:

where the subscripts 1 and 2 indicate the first and second halves of the data, *F* denotes Fourier coefficients, subscript T is for Fourier coefficients from ‘true’ images and *n* denotes the noise. We also assume that the noise has zero mean, and that var(*n*_1_) = var(*n*_2_). As a consequence, var(*n*) = var(*n*_1_)/2. Under the assumptions stated above,

This can be expressed in terms of the ratio of the variance of the noise to the variance of the signal,

Now let us consider Fourier shell correlation between two full hypothetical data sets with Fourier coefficients *F*_1full_ and *F*_2full_. In this case

Finally, we calculate the correlation between a full data reconstructed map and the ‘true’ map,

To clarify, the FSC_1/2_ used in this paper is the Fourier shell correlation between half-data reconstructions cor(*F*_1_, *F*_2_).

## References

[bb2] Afonine, P. V., Grosse-Kunstleve, R. W., Urzhumtsev, A. & Adams, P. D. (2009). *J. Appl. Cryst.* **42**, 607–615. 10.1107/S0021889809023528 PMC2712840 19649324

[bb3] Afonine, P. V., Headd, J. J., Terwilliger, T. C. & Adams, P. D. (2013). *Comput. Crystallogr. Newsl.* **4**, 43–44. https://www.phenix-online.org/newsletter/CCN_2013_07.pdf.

[bb4] Afonine, P. V., Moriarty, N. W., Mustyakimov, M., Sobolev, O. V., Terwilliger, T. C., Turk, D., Urzhumtsev, A. & Adams, P. D. (2015). *Acta Cryst.* D**71**, 646–666. 10.1107/S1399004714028132 PMC4356370 25760612

[bb5] Afonine, P. V., Poon, B. K., Read, R. J., Sobolev, O. V., Terwilliger, T. C., Urzhumtsev, A. & Adams, P. D. (2018). *Acta Cryst.* D**74**, 531–544. 10.1107/S2059798318006551 PMC6096492 29872004

[bb7] Bartesaghi, A., Merk, A., Banerjee, S., Matthies, D., Wu, X., Milne, J. L. & Subramaniam, S. (2015). *Science*, **348**, 1147–1151. 10.1126/science.aab1576 PMC6512338 25953817

[bb8] Berman, H. M. *et al.* (2002). *Acta Cryst.* D**58**, 899–907. 10.1107/s0907444902003451 12037327

[bb9] Brown, A., Long, F., Nicholls, R. A., Toots, J., Emsley, P. & Murshudov, G. (2015). *Acta Cryst.* D**71**, 136–153. 10.1107/S1399004714021683 PMC4304694 25615868

[bb10] Brunger, A. T., DeLaBarre, B., Davies, J. M. & Weis, W. I. (2009). *Acta Cryst.* D**65**, 128–133. 10.1107/S0907444908043795 PMC2631637 19171967

[bb11] Burnley, T., Palmer, C. M. & Winn, M. (2017). *Acta Cryst.* D**73**, 469–477. 10.1107/S2059798317007859 PMC5458488 28580908

[bb12] Casañal, A., Kumar, A., Hill, C. H., Easter, A. D., Emsley, P., Degliesposti, G., Gordiyenko, Y., Santhanam, B., Wolf, J., Wiederhold, K. & Dornan, G. L. (2017). *Science*, 1056–1059. 10.1126/science.aao6535 PMC5788269 29074584

[bb13] Cowtan, K. (2003). *IUCr Comput. Commun. Newsl.* **2**, 4–9. https://www.iucr.org/resources/commissions/crystallographic-computing/newsletters/2.

[bb14] Emsley, P., Lohkamp, B., Scott, W. G. & Cowtan, K. (2010). *Acta Cryst.* D**66**, 486–501. 10.1107/S0907444910007493 PMC2852313 20383002

[bb15] Faruqi, A. R. & McMullan, G. (2011). *Q. Rev. Biophys.* **44**, 357–390. 10.1017/S0033583511000035 21524337

[bb16] Gong, Z., Schwieters, C. D. & Tang, C. (2015). *PLoS One*, **10**, e0120445. 10.1371/journal.pone.0120445 PMC4370883 25798848

[bb17] Grigorieff, N. (2007). *J. Struct. Biol.* **157**, 117–125. 10.1016/j.jsb.2006.05.004 16828314

[bb18] Headd, J. J., Echols, N., Afonine, P. V., Grosse-Kunstleve, R. W., Chen, V. B., Moriarty, N. W., Richardson, D. C., Richardson, J. S. & Adams, P. D. (2012). *Acta Cryst.* D**68**, 381–390. 10.1107/S0907444911047834 PMC3322597 22505258

[bb19] Headd, J. J., Echols, N., Afonine, P. V., Moriarty, N. W., Gildea, R. J. & Adams, P. D. (2014). *Acta Cryst.* D**70**, 1346–1356. 10.1107/S1399004714003277 PMC4014122 24816103

[bb20] Heel, M. van & Schatz, M. (2017). *bioRxiv*, 224402. https://doi.org/10.1101/224402.

[bb21] Huber, P. J. (2011).* International Encyclopedia of Statistical Science*, edited by M. Lovirc, pp. 1248–1251. Berlin, Heidelberg: Springer.

[bb22] Jakobi, A. J., Wilmanns, M. & Sachse, C. (2017). *eLife*, **6**, e27131. 10.7554/eLife.27131 PMC5679758 29058676

[bb23] Joseph, A. P., Malhotra, S., Burnley, T., Wood, C., Clare, D. K., Winn, M. & Topf, M. (2016). *Methods*, **100**, 42–49. 10.1016/j.ymeth.2016.03.007 PMC4854230 26988127

[bb24] Karplus, P. A. & Diederichs, K. (2012). *Science*, **336**, 1030–1033. 10.1126/science.1218231 PMC3457925 22628654

[bb25] Kirkland, E. J. (2010). *Advanced Computing in Electron Microscopy.* New York: Springer.

[bb26] Kirmizialtin, S., Loerke, J., Behrmann, E., Spahn, C. M. & Sanbonmatsu, K. Y. (2015). *Methods Enzymol.* **558**, 497–514. 10.1016/bs.mie.2015.02.011 26068751

[bb27] Kostelec, P. J. & Rockmore, D. N. (2007). *SOFT: SO(3) Fourier Transforms*. https://www.cs.dartmouth.edu/~geelong/soft/.

[bb28] Kovalevskiy, O., Nicholls, R. A., Long, F., Carlon, A. & Murshudov, G. N. (2018). *Acta Cryst.* D**74**, 215–227. 10.1107/S2059798318000979 PMC5947762 29533229

[bb29] Kucukelbir, A., Sigworth, F. J. & Tagare, H. D. (2014). *Nature Methods*, **11**, 63–65. 10.1038/nmeth.2727 PMC3903095 24213166

[bb30] Kühlbrandt, W. (2014). *Elife*, **3**, e03678. 10.7554/eLife.03678 PMC4131193 25122623

[bb31] Long, F., Nicholls, R. A., Emsley, P., Gražulis, S., Merkys, A., Vaitkus, A. & Murshudov, G. N. (2017). *Acta Cryst.* D**73**, 112–122. 10.1107/S2059798317000067 PMC5297914 28177307

[bb32] Lyumkis, D., Brilot, A. F., Theobald, D. L. & Grigorieff, N. (2013). *J. Struct. Biol.* **183**, 377–388. 10.1016/j.jsb.2013.07.005 PMC3824613 23872434

[bb33] McCoy, A. J., Grosse-Kunstleve, R. W., Adams, P. D., Winn, M. D., Storoni, L. C. & Read, R. J. (2007). *J. Appl. Cryst.* **40**, 658–674. 10.1107/S0021889807021206 PMC2483472 19461840

[bb34] Murshudov, G. N. (2016). *Methods Enzymol.* **579**, 277–305. 10.1016/bs.mie.2016.05.033 27572731

[bb35] Murshudov, G. N., Skubák, P., Lebedev, A. A., Pannu, N. S., Steiner, R. A., Nicholls, R. A., Winn, M. D., Long, F. & Vagin, A. A. (2011). *Acta Cryst.* D**67**, 355–367. 10.1107/S0907444911001314 PMC3069751 21460454

[bb36] Navaza, J. (1994). *Acta Cryst.* A**50**, 157–163.

[bb37] Nicholls, R. A., Fischer, M., McNicholas, S. & Murshudov, G. N. (2014). *Acta Cryst.* D**70**, 2487–2499. 10.1107/S1399004714016241 PMC4157452 25195761

[bb38] Nicholls, R. A., Kovalevskiy, O. & Murshudov, G. N. (2017). *Methods Mol Biol.* **1607**, 565–593. 10.1007/978-1-4939-7000-1_23 28573589

[bb39] Nicholls, R. A., Long, F. & Murshudov, G. N. (2012). *Acta Cryst.* D**68**, 404–417. 10.1107/S090744491105606X PMC3322599 22505260

[bb40] Nicholls, R. A., Long, F. & Murshudov, G. N. (2013). *Advancing Methods for Biomolecular Crystallography*, edited by R. Read, A. Urzhumtsev & V. Y. Lunin, pp. 231–258. Dordrecht: Springer.

[bb41] Pellegrini, L., Yu, D. S., Lo, T., Anand, S., Lee, M., Blundell, T. L. & Venkitaraman, A. R. (2002). *Nature (London)*, **420**, 287–293. 10.1038/nature01230 12442171

[bb42] Peng, L.-M., Ren, G., Dudarev, S. L. & Whelan, M. J. (1996). *Acta Cryst.* A**52**, 257–276.

[bb43] Pettersen, E. F., Goddard, T. D., Huang, C. C., Couch, G. S., Greenblatt, D. M., Meng, E. C. & Ferrin, T. E. (2004). *J. Comput. Chem.* **25**, 1605–1612. 10.1002/jcc.20084 15264254

[bb44] Rawi, R., Whitmore, L. & Topf, M. (2010). *Bioinformatics*, **26**, 1673–1674. 10.1093/bioinformatics/btq237 PMC2887048 20444836

[bb45] Roseman, A. M. (2000). *Acta Cryst.* D**56**, 1332–1340. 10.1107/s0907444900010908 10998630

[bb46] Rosenthal, P. B. & Henderson, R. (2003). *J. Mol. Biol.* **333**, 721–745. 10.1016/j.jmb.2003.07.013 14568533

[bb47] Scheres, S. H. W. (2012). *J. Struct. Biol.* **180**, 519–530. 10.1016/j.jsb.2012.09.006 PMC3690530 23000701

[bb48] Scheres, S. H. W. (2014). *Elife*, **3**, e03665. 10.7554/eLife.03665 PMC4130160 25122622

[bb49] Schröder, G. F., Brunger, A. T. & Levitt, M. (2007). *Structure*, **15**, 1630–1641. 10.1016/j.str.2007.09.021 PMC2213367 18073112

[bb50] Schröder, G. F., Levitt, M. & Brunger, A. T. (2010). *Nature (London)*, **464**, 1218–1222. 10.1038/nature08892 PMC2859093 20376006

[bb52] Settembre, E. C., Chen, J. Z., Dormitzer, P. R., Grigorieff, N. & Harrison, S. C. (2011). *EMBO J.* **30**, 408–416. 10.1038/emboj.2010.322 PMC3025467 21157433

[bb53] Sheldrick, G. M. (2015). *Acta Cryst.* C**71**, 3–8.

[bb54] Short, J. M., Liu, Y., Chen, S., Soni, N., Madhusudhan, M. S., Shivji, M. K. & Venkitaraman, A. R. (2016). *Nucleic Acids Res.* **44**, 9017–9030. 10.1093/nar/gkw783 PMC5100573 27596592

[bb55] Skubák, P. & Pannu, N. S. (2013). *Nature Commun.* **4**, 2777. 10.1038/ncomms3777 PMC3868232 24231803

[bb56] Smart, O. S., Womack, T. O., Flensburg, C., Keller, P., Paciorek, W., Sharff, A., Vonrhein, C. & Bricogne, G. (2012). *Acta Cryst.* D**68**, 368–380. 10.1107/S0907444911056058 PMC3322596 22505257

[bb57] Sun, L., Zhang, X., Gao, S., Rao, P. A., Padilla-Sanchez, V., Chen, Z., Sun, S., Xiang, Y., Subramaniam, S., Rao, V. B. & Rossmann, M. G. (2015). *Nature Commun.* **6**, 7548. 10.1038/ncomms8548 PMC4493910 26144253

[bb58] Terwilliger, T. C., Read, R. J., Adams, P. D., Brunger, A. T., Afonine, P. V. & Hung, L.-W. (2013). *Acta Cryst.* D**69**, 2244–2250. 10.1107/S0907444913017770 PMC3817698 24189236

[bb59] Terwilliger, T. C., Sobolev, O. V., Afonine, P. V. & Adams, P. D. (2018). *Acta Cryst.* D**74**, 545–559. 10.1107/S2059798318004655 PMC6096490 29872005

[bb60] Trabuco, L. G., Villa, E., Mitra, K., Frank, J. & Schulten, K. (2008). *Structure*, **16**, 673–683. 10.1016/j.str.2008.03.005 PMC2430731 18462672

[bb61] Trabuco, L. G., Villa, E., Schreiner, E., Harrison, C. B. & Schulten, K. (2009). *Methods*, **49**, 174–180. 10.1016/j.ymeth.2009.04.005 PMC2753685 19398010

[bb62] Vagin, A. & Teplyakov, A. (2010). *Acta Cryst.* D**66**, 22–25. 10.1107/S0907444909042589 20057045

[bb63] Wang, R. Y.-R., Song, Y., Barad, B. A., Cheng, Y., Fraser, J. S. & DiMaio, F. (2016). *Elife*, **5**, e17219. 10.7554/eLife.17219 PMC5115868 27669148

[bb64] Winn, M. D. *et al.* (2011). *Acta Cryst.* D**67**, 235–242. 10.1107/S0907444910045749 PMC3069738 21460441

[bb65] Wlodawer, A. & Dauter, Z. (2017). *Acta Cryst.* D**73**, 379–380. 10.1107/S205979831700225X PMC5379937 28375149

[bb66] Wlodawer, A., Li, M. & Dauter, Z. (2017). *Structure*, **25**, 1589–1597. 10.1016/j.str.2017.07.012 PMC5657611 28867613

[bb67] Wood, C., Burnley, T., Patwardhan, A., Scheres, S., Topf, M., Roseman, A. & Winn, M. (2015). *Acta Cryst.* D**71**, 123–126. 10.1107/S1399004714018070 PMC4304692 25615866

[bb68] Zhang, F., Chen, Y., Ren, F., Wang, X., Liu, Z. & Wan, X. (2017). *IEEE/ACM Trans. Comput. Biol. Bioinform.* **14**, 316–325. 10.1109/TCBB.2015.2415787 28368809

